# Reorganization of Muscle Coordination Underlying Motor Learning in Cycling Tasks

**DOI:** 10.3389/fbioe.2020.00800

**Published:** 2020-07-15

**Authors:** Diego Torricelli, Cristiano De Marchis, Andrea d’Avella, Daniel Nemati Tobaruela, Filipe Oliveira Barroso, Jose L. Pons

**Affiliations:** ^1^Neural Rehabilitation Group, Cajal Institute, Spanish National Research Center (CSIC), Madrid, Spain; ^2^Biomedical Engineering Laboratory, Department of Engineering, Università Roma TRE, Rome, Italy; ^3^Laboratory of Neuromotor Physiology, IRCCS Fondazione Santa Lucia, Rome, Italy; ^4^Department of Biomedical and Dental Sciences and Morphofunctional Imaging, Università di Messina, Messina, Italy; ^5^Legs and Walking Lab, Shirley Ryan AbilityLab (formerly Rehabilitation Institute of Chicago), Chicago, IL, United States; ^6^Department of Physical Medicine and Rehabilitation, Feinberg School of Medicine, Northwestern University, Chicago, IL, United States; ^7^Department of Biomedical Engineering, McCormick School of Engineering and Applied Science, Northwestern University, Chicago, IL, United States; ^8^Department of Mechanical Engineering, McCormick School of Engineering and Applied Science, Northwestern University, Chicago, IL, United States

**Keywords:** muscle synergies, pedaling, adaptation, modular control, EMG biofeedback

## Abstract

The hypothesis of modular control, which stands on the existence of muscle synergies as building blocks of muscle coordination, has been investigated in a great variety of motor tasks and species. Yet, its role during learning processes is still largely unexplored. To what extent is such modular control flexible, in terms of spatial structure and temporal activation, to externally or internally induced adaptations, is a debated issue. To address this question, we designed a biofeedback experiment to induce changes in the timing of muscle activations during leg cycling movements. The protocol consisted in delaying the peak of activation of one target muscle and using its electromyography (EMG) envelope as visual biofeedback. For each of the 10 healthy participants, the protocol was repeated for three different target muscles: Tibialis Anterioris (TA), Gastrocnemius Medialis (GM), and Vastus Lateralis (VL). To explore the effects of the conditioning protocol, we analyzed changes in the activity of eight lower limb muscles by applying different models of modular motor control [i.e., fixed spatial components (FSC) and fixed temporal components (FTC)]. Our results confirm the hypothesis that visual EMG biofeedback is able to induce changes in muscle coordination. Subjects were able to shift the peak of activation of the target muscle, with a delay of (49 ± 27°) across subjects and conditions. This time shift generated a reorganization of all the other muscles in terms of timing and amplitude. By using different models of modular motor control, we demonstrated that neither spatially invariant nor temporally invariant muscle synergies alone were able to account for these changes in muscle coordination after learning, while temporally invariant muscle synergies with adjustments in timing could capture most of muscle activity adaptations observed after the conditioning protocol. These results suggest that short-term learning in rhythmic tasks is built upon synergistic temporal commands that are robust to changes in the task demands.

## Introduction

Understanding how the central nervous system (CNS) orchestrates muscle coordination is a fundamental step to deepen our knowledge in the mechanisms underlying movement generation, motor skill acquisition, and motor adaptation to externally induced perturbation. According to the hypothesis of muscle synergies, the CNS manages muscle redundancy by means of functional units, namely, muscle synergies or modules, which are recruited in time by a reduced set of activation signals (Torricelli et al., 2016). In the last 20 years, increasing experimental evidence has been supporting this hypothesis for a great variety of motor tasks (Ivanenko et al., 2004; Clark et al., 2010; Gonzalez-Vargas et al., 2015).

Motor adaptation and learning have been widely studied in humans by means of computational models, with the aim of describing the modification of the internal models to new or changing environments (Krakauer and Mazzoni, 2011; Wolpert et al., 2011). The muscle synergies framework has been recently proposed as a general model for describing these processes under the muscle coordination point of view. Berger et al. (2013) provided evidence for the modular organization of motor control using a virtual upper limb reaching task paradigm, showing that adaptation to rotations in the force fields that are incompatible with previously acquired modular structures led to significantly lower learning rates. This study highlighted the existence of a flexible structure upon which fast adaptation was achieved by tuning the recruitment of fixed modules. Sawers et al. (2015) have explored the acquisition of new motor behaviors, showing that more complex skills are typically associated with a higher number of modules. De Marchis et al. (2013b) have explored the short-term learning mechanisms in a novel pedaling paradigm using visual biofeedback of pedal force. They showed that short-term motor learning could be accounted for by the use of baseline synergies plus a few additional ones. Jacobs et al. (2018) analyzed the re-organization of muscle coordination during adaptation to walking in a powered ankle exoskeleton. They showed that subjects adapted the temporal activation patterns during the adaptation phase, keeping unaltered the pre-existing synergies both in number and spatial composition. Modular motor control models have also been explored during visuomotor adaptation tasks, highlighting that a complete adaptation to visuo-motor distortions can be achieved by tuning the recruitment of a set of fixed spatial synergies. Gentner et al. (2013) proved that adaptation to a 45° visuo-motor rotation, applied during upper limb isometric virtual reaching tasks, was reached after few trials through a rotation of the recruitment of a set of fixed baseline spatial muscle synergies. A similar experiment by De Marchis et al. (2018) explored whether the same adaptation mechanism was present when the visuo-motor perturbation was applied to only a portion of the workspace, highlighting that a different recruitment of the same baseline spatial synergies led to the same full biomechanical adaptation when the order of the perturbations was changed.

Most of these studies hypothesized a unique control model based on the temporal tuning of a set of spatially fixed muscle synergies within the synchronous muscle synergies model. To our knowledge, no study has explored the possibility of using alternative models to explain the neuromotor reorganization, e.g., assuming invariant temporal activation patterns, time-varying muscle synergies, and space-by-time synergies (d’Avella et al., 2003; Ivanenko et al., 2005; Cappellini et al., 2006; Delis et al., 2018). In our opinion, exploring these models is a necessary step to identify those which can better describe the effects of learning/adaptation processes on muscle coordination in both subject- and task-specific way (Safavynia and Ting, 2012).

**FIGURE 1:**
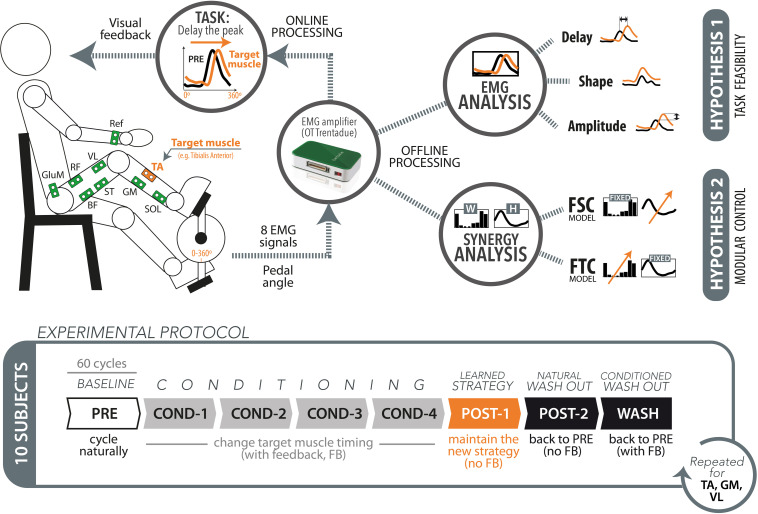
Schematic representation of the experimental setup, protocol, and data analysis.

In this work, we investigated the effects on muscle coordination when learning new pedaling tasks. We asked subjects to accomplish one functional goal: changing the activation timing of one target muscle. To do so it is necessary to alter the usual muscle activation pattern for pedaling, therefore requiring a learning/adaptation mechanism to generate muscle patterns with the novel activation timing yet still capable of accomplishing the task in a functional way. Subjects were provided with a visual feedback of the sEMG envelope of one target muscle at the end of each pedaling cycle. This was used as a visual representation of an internal variable that is directly related to the motor output and indirectly representative of the underlying motor control strategies. The experiment was designed to test two main hypotheses (see Figure 1):

(1) Task feasibility hypothesis: The paradigm based on electromyography (EMG) timing biofeedback can successfully lead to the desired changes in the target muscle timing. This hypothesis stands on the assumption that the required changes in muscle activation are biomechanically compatible with the execution of the highly constrained pedaling task, and we here test the hypothesis that this kind of visual feedback can be used by the subject for changing the EMG timing in real time.
(2) Modular control hypothesis: The changes produced on the target muscle are propagated to the other muscles, but this change in muscle coordination does not imply a change of the underlying modular structures. This hypothesis stands on the assumption that short-term learning/adaptation processes do not affect the existing modular control schemes, as supported by previous studies. We tested the following two complementary models:
  (a) Fixed spatial components (FSC) model: The changes in muscle coordination result from time invariant and spatially fixed synergy vectors (spatial synergies, W_*FSC*_) with flexible temporal activation coefficients [H_*FSC*_(t)].
  (b) Fixed temporal components (FTC) model: The changes in muscle coordination result from invariant temporal components (temporal synergies, H_*FTC*_) with flexible synergy vectors varying cycle-by-cycle [W_*FTC*_(*t*)].

## Materials and Methods

### Experimental Protocol

Ten healthy subjects (two females and eight males), without any known motor or neurological lesions, participated in the experiment. Subject’s age was 25.1 ± 4.4 years (mean ± SD). The experiment was done in the facilities of the Neural Rehabilitation Group of the Cajal Institute (Madrid, Spain), Spanish National Research Council (CSIC). The experimental procedures were approved by the Bioethical subcommittee of the Ethical committee of CSIC (Spanish National Research Council, reference 008/2016). The whole study was in accordance with the principles of the Declaration of Helsinki.

Prior to the experiment, surface Ag/AgCl EMG electrodes (TenderTrodeTM, Vermed, United States) were placed on eight muscles of the subject’s dominant leg: Tibialis Anterioris (TA), Soleus (SOL), Gastrocnemius Medialis (GM), Semitendinous (ST), Biceps Femoris (BF), Vastus Lateralis (VL), Rectus Femoris (RF), and Gluteus Medius (GluM). A reference electrode was located on the wrist, in the same side of the dominant leg. Such location was selected due to the lack of muscle activity and no observable movement of the joint or cables during the experiment. The electrodes were placed according to SENIAM recommendations (Hermens, 2000). Adhesive tape was used to fix the cables to the skin to minimize movement-induced artifacts. The EMG activity was recorded at a sample frequency of 1000 Hz using the wireless EMG amplifier “Trentadue” (OT Bioelettronica, Torino, Italy). The appropriate placement of the electrodes was verified by visually checking the resulting EMG signals through the software interface.

Participants were sat on a chair and asked to pedal on a recumbent cycling ergometer (MOTOmed VIVA2, Reck, Germany) while looking at a computer screen displaying the muscle activity of one muscle of the subject. Prior to the experiment, both feet were fastened to the pedals of the ergometer by means of straps provided by the system for this purpose. The resistive load of the bicycle was adjusted to ensure visible muscle activity during pedaling. The subject sat on a chair in a comfortable position, with the trunk approximately vertical and leaning on the backrest, while the hands were placed on the handle of the ergometer.

The experiment consisted on three consecutive sessions, each composed of seven cycling trials of 60 cycles each (see Figure 1, lower panel). The first trial, named “PRE,” was used to obtain a reference of muscle activity during self-selected speed to be used afterward, during the biofeedback trials. After the PRE trial, the subjects performed four consecutive trials in which they received visual feedback on the EMG envelope of one muscle, called *target* muscle. In these conditioning trials, labeled with the prefix “COND,” the subjects were asked to delay as much as possible the peak of muscle activity with respect to the reference muscle activity recorded during the PRE trial. The visual feedback was provided at the end of each pedaling cycle. No specific indications on how to achieve the task goal were given, allowing the subjects to freely find their own neuromuscular strategy. After the four conditioning trials, we tested the after-effects of the conditioning experiment by means of two “POST” trials. In the POST-1 trial, the subjects were asked to maintain the new strategy learned during the four conditioning trials, but in the absence of visual feedback. This trial has been designed to test whether the subjects actually learned the conditioning process induced by the visual feedback. In the POST-2 trial, also without visual feedback, the participants were asked to perform a normal pedaling movement, as done in the PRE trial. This trial has been conceived to test the permanence of involuntary after-effects. A final trial (WASH) was executed to ensure the elimination of any residual effect of the trial on muscle activation, and to prepare for the next experimental session. In this trial the subject was asked to return to baseline (PRE) muscle patterns, with the help of visual feedback.

The aforementioned protocol was executed three times, each with a different target muscle. We chose the three target muscles TA, VL, and GM, being them the dominant muscles for the three muscle synergies found during pedaling tasks (Barroso et al., 2014), i.e., presenting higher weight in one synergy and very low weight in the other two synergies. A 2-min rest between trials and a 5-min rest between sessions were used to avoid muscle fatigue. The experiment lasted approximately 2 h per participant, including donning and doffing. To measure the pedaling angle, we integrated a custom-made magnetic encoder in the crank of the ergometer, synchronized with the EMG amplifier and the processing software. The encoder was calibrated in such a way to obtain a 0° angle when the crank was in the bottom dead center. The synchronized acquisition of EMG from the Trentadue Amplifier, as well as the post processing was implemented in Matlab^®^ 2010a.

### Data Analysis

#### EMG Pre-processing

The raw EMG signal from all muscles was pre-processed online at the end of each pedaling cycle, defined as the crank positioned in the bottom dead center, pointing toward the ground (see Figure 1). We used a second-order Butterworth bandpass filter at 20–400 Hz to filter low-frequency motion artifacts and high-frequency electromagnetic noise (Raez et al., 2006). We applied a full-wave rectification and a low-pass filtering at 4 Hz to obtain the basic set of EMG envelopes from each cycle. The corresponding raw EMG data were stored for subsequent offline processing. At the same time, the EMG envelope of the target muscle was normalized in amplitude and displayed immediately after each cycle to the user. To prepare for offline processing, the set of non-normalized EMG envelopes obtained from each cycle were amplitude-normalized to the median peak value across the 60 cycles of the PRE trial, then time-normalized on a 1-by-360 vector, and finally concatenated to obtain a 8-by-21,600 matrix (M) of muscle envelopes (Hug et al., 2011).

#### Individual EMG Analysis

To test the first hypothesis (i.e., *task feasibility*), we analyzed the individual changes in activation timing, shape, and amplitude of the target muscles independently (see Figure 1, top panel). The timing analysis was performed using circular statistics (Batschelet, 1981; Fisher, 1996) on the population of the peaks of the EMG envelopes of each trial. We computed the mean direction for circular data for each population of 60-peak timings, according to the following equations:

(1) $$A=\sum_{i=1}^{60}\text{cos}\theta_{i}$$

(2) $$B=\sum_{i=1}^{60}\text{sin}\theta_{i}$$

(3) $$\bar{\theta }=tan^{-1}\left(\frac{B}{A}\right)$$

where θ_*i*_ represents the timing of the peak of a single cycle, expressed in radians, and $\bar{\theta }$ is the resulting mean direction. Delay was calculated as the difference between the mean direction of each trial and the mean direction of the PRE trial.

The shape similarity (SS) was computed by applying a circular cross correlation *C*_*xy*_, as described by the following equation:

(4) $$SS_{xy}=\frac{C_{xy}}{\sqrt{\sum_{i=1}^{360}x_{i}^{2}\sum_{i=1}^{360}y_{i}^{2}}}$$

where *C*_*xy*_ is the non-normalized circular cross-correlation at lag zero, *x* denotes the EMG envelope of the current cycle, and *y* denotes the mean EMG envelope of the PRE trial.

The amplitude analysis was performed by calculating the difference in amplitude between each peak and the PRE trial, according to the following equation:

(5) $$A_{x}=\frac{max\left\{x\right\}-max\left\{y\right\}}{max\left\{y\right\}}$$

where *x* is the EMG envelope of the current cycle and *y* is the mean envelope of the PRE trial.

#### Muscle Synergy Analysis

To test the second hypothesis (i.e., *modular control*), we assessed the ability of the FSC and FTC models to explain the variance of the measured EMG. The FSC model had fixed synergy vectors (W) and variable activation coefficients (H). The activation of the *k*th muscle at time *t* can be defined according to the following formulation:

(6) $$M_{kt}=\sum_{i=1}^{S}W_{ki}H_{it}$$

where *S* is the number of synergies and *T* is the number of time points. In this model, the fixed structure is constituted by the spatial synergies W, a K times S matrix named W_*FSC*_ from now on, while H is continuously varying in time.

The FTC model had fixed H and variable W, and the activation of the *k*th muscle, at the *n*th sample of the *c*th pedaling cycle, can be described as in the following:

(7) $${\left(M_{kn}\right)}_{c}=\sum_{i=1}^{S}{\left(W_{ki}\right)}_{c}H_{in}$$

in this latter model the fixed component is constituted by the temporal synergies H, an S times N matrix named H_*FTC*_ from now on, while W varies from cycle to cycle.

**FIGURE 2:**
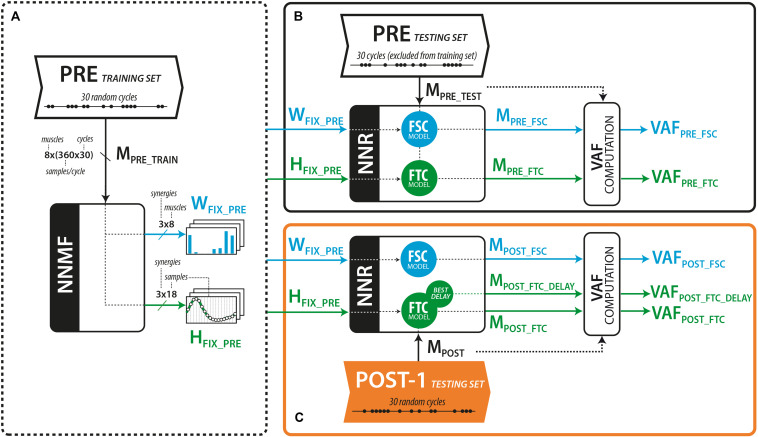
**(A)** Calculation of the muscle synergy vector (W) and activation coefficients (H) from the PRE training set. **(B)** Reconstruction of the EMG envelopes (M) from the PRE trial, considering the cycles excluded from the training set. **(C)** Reconstruction of the EMG envelopes (M) from the POST-1 testing set, using fixed W (FSC model) or fixed H (FTC model). An extended version of the FTC model consisted in shifting the H vectors until the best VAF is obtained.

For both models (see Figure 2) the 60 pedaling cycles of each trial were split into a 30-cycle training set, and a 30-cycle testing set. The 30 cycles were randomly selected from the 60-cycle pool. For each trial of each subject, the training set was used to extract the muscle synergy vectors W_*FSC*_ (FSC model) or the temporal components H_*FTC*_ (FTC model) using the standard non-negative matrix factorization (NNMF) algorithm (Lee and Seung, 1999). The testing set was used to obtain the matrix of reconstructed EMG (M_*REC*_) using non-negative reconstruction (NNR). NNR consists in the application of the standard NNMF algorithm, either by fixing spatial component (W) extracted from the training set and varying the matrix of activation coefficients (H), or vice versa, according to the following multiplicative update rule (the equation below considers the case of fixed W and varying H):

(8) $$H_{rc}=H_{rc}\frac{{\left(W_{PRE}^{T}M\right)}_{rc}}{{\left(W_{PRE}^{T}W_{PRE}H\right)}_{rc}}$$

where *r* (row) and *c* (column) denote the single components of the matrices taken into account, T denotes the transposed matrix, and H is the reconstructed activation coefficients matrix (H_*REC*_).

For both FSC and FTC models, the number of synergies *S* was chosen as the smallest number able to exceed the 90% of variance accounted for (VAF) (Clark et al., 2010) on synergies extracted from the training set. In order to partially compensate for potential effects of the different number of degrees of freedom between the two models, we performed the whole analysis by down-sampling the original data so to obtain H and W data matrices with dimension *N*-by-*S*, being *N* = 8 the number of muscles in the FSC model and *N* = 18 the number of time points in the FTC model (chosen as the minimum number of points able to preserve the shape of the temporal commands). In this way, we obtained a comparable number of degrees of freedom in the sub-dimensional structures in the two models.

##### Fixed spatial components (FSC) model

To test the FSC model (see Figure 2), we applied NNR using the fixed set of muscle synergies extracted from the PRE training set (W_*FSC_PRE*_, see Figure 2A) and updated H at every algorithm iteration to obtain the reconstructed muscle activations from the testing sets of both PRE (M_*PRE_FSC*_, see Figure 2B) and POST-1 (M_*POST_FSC*_, see Figure 2C) trials. The goodness of the reconstruction with W_*FSC_PRE*_ was assessed with the VAF resulting from the application of NNR.

##### Fixed temporal components (FTC) model

To test the FTC model, we applied NNR using the set of temporal components H_*FTC_PRE*_ extracted from the PRE training set (Figure 2A) to reconstruct the testing set of the PRE and POST-1 trial, respectively (Figures 2B,C). We applied NNR as done for the FSC model, but maintaining H fixed and updating W at every algorithm iteration, obtaining the reconstructed muscle activations M_*PRE_FTC*_ and M_*POST_FTC*_, respectively. An extended version of the FTC model (M_*POST_FTC_DELAY*_) was further tested by reconstructing M_*POST*_ with all the possible time shifted versions of the components of H_*FSC_PRE*_ (FTC_*DELAY*_) independently. This approach allowed us to test whether the muscle coordination in POST-1 was explained by a simple time shift of the original components (i.e., without alterations in the shape of the temporal commands). In this latter model, an optimal set of delays of the temporal components was found as the one leading to the higher reconstruction VAF of the POST-1 trial.

##### FSC and FTC models validation

In order to validate the consistency of the tested models (FSC, FTC, and FTC_*DELAY*_), we built a surrogate dataset to define the statistical level of chance when fitting the EMG data. For the FSC model, for each subject and trial, we applied NNR to a surrogate version of the extracted W_*FSC_PRE*_, constructed by randomly shuffling the muscle components of each synergy vector of W_*FSC_PRE*_. This surrogate version of the synergy matrix corresponds to an anatomical disruptor, leading to synergy vectors that only maintain their Euclidean norm with respect to the original ones. For each subject, trial, and biofeedback condition, 100 reconstructions of M_*POST*_ via NNR were applied with 100 different surrogate versions of W_*FSC_PRE*_ (W_*SURR*_), leading to 100 VAF values expected from unstructured synergy vectors.

The surrogate data analysis for the FTC model was performed by constructing a Fourier based surrogate version of the temporal commands H_*FTC_PRE*_. A Fourier transform was applied to each H_*FTC_PRE*_ component (Matlab “fft” function), and a surrogate version in the frequency domain was built by randomly shuffling the phase components of the Fourier transform, while keeping its modulus unaltered (Faes et al., 2004). The surrogate version of the temporal-component matrix was then calculated by applying the inverse Fourier transform, in order to obtain a temporal command with the same modulus of the Fourier transform, but shuffled phases. This led to temporal components with an altered morphology and main peak position in the time domain, induced by the phase shuffling. For each subject, trial and biofeedback condition, 100 reconstructions of M_*POST*_ via NNR were applied with 100 different surrogate versions of H_*FTC_PRE*_ (H_*SURR*_) leading to 100 VAF values expected from phase distorted temporal commands.

The surrogate data analysis for the FTC_*DELAY*_ model was carried out in the same way as for the FTC model, with the only difference that, for each subject, biofeedback condition and synergy, each of the 100 surrogate versions of H_*FTC_PRE*_ was time shifted by all the possible time-shifts along the pedaling cycle (H_*SURR_DELAY*_). In this way, we checked whether a potential good reconstruction of M_*POST*_ via the FTC_*DELAY*_ model could derive from a probable shape matching of a quantity with the same frequency content with respect to the original one (i.e., there is a high probability that among all the possible shifts of the surrogate temporal commands, some of them lead to a good reconstruction of M_*POST*_).

For the previously described approaches to build surrogate data W_*SURR*_ and H_*SURR*_ for each subject, trial and biofeedback condition, the significance threshold VAF_*TH_SURR*_ level was set as the 95th percentile of all the obtained surrogate VAF values. For the FTC_*DELAY*_ model, this significance threshold was calculated over the set of the best performing H_*SURR_DELAY*_ for each subject and biofeedback condition, corresponding to the delay leading to the highest reconstruction VAF.

#### Statistical Analysis

A PRE-POST comparison was carried out in terms of pedaling cadence (PC) for the three biofeedback conditions (TA, VL, and GM), by using a paired Wilcoxon signed rank test, in order to check whether any difference in muscle activation and timing could be ascribed to a mismatch in PC.

For each subject, the significance of time delays with respect to the PRE trial has been tested applying the Watson–Williams test for circular data (Fisher, 1996). The same test was used to verify the similarity of time delays across all subjects, for each muscle and feedback session. Circular statistics were performed using the Circular Statistics Matlab Toolbox (Berens, 2009). To test the significance of the difference in amplitude of the EMG peaks between each trial and the PRE trial, we used a paired-sample *t*-test. The circular cross correlation with respect to the PRE trial was tested with the Mann–Whitney–Wilcoxon test (Matlab function “ranksum”). This test was applied after checking the non-normality of these distributions. To assess the significance of the VAF values obtained via NNR on the PRE and POST-1 trials and those obtained using the surrogate data, a Wilcoxon signed rank test was applied. The test was used to compare the obtained VAF_*REC_PRE*_, VAF_*REC_POST*_, and VAF_*TH_SURR*_ values for each subject, biofeedback condition, and trial. The significance level of the *p*-value has been set to 0.05 in all aforementioned tests.

Moreover, for each biofeedback condition (TA, VL, and GM), VAF reconstruction values emerging from the different models were compared among trials using ANOVA test with models (FSC_*PRE*_, FTC_*PRE*_, FSC_*POST*_, FTC_*POST*_, and FTC_*POST*___*DELAY*_) as factors. In case of significant effect, *post hoc* analysis was carried out with Bonferroni correction.

We performed an additional PRE–POST-1 comparison of the modular structures emerging from the FSC and FTC models (i.e., time varying cycle-by-cycle H for the FSC and cycle-by-cycle varying W for the FTC). The H emerging from the FSC models were compared in terms of circular cross-correlation, as explained in Section “Individual EMG Analysis” for the single EMG data, so as to obtain an SS index and a delay. The W emerging from the FTC model were compared between PRE and POST-1 in terms of normalized scalar product to check their similarity. A further comparison between the FSC and the FTC models was conducted on the emerging synergy matrices W (fixed in the FSC model and varying in the FTC model), in order to check whether similar spatial muscle synergies act as a base for the different modular control models. This comparison was carried out in terms of cosine similarity between homologous pairs of synergies.

## Results

No statistically significant difference in PC was observed between the PRE and POST-1 trials for all the analyzed biofeedback conditions (PC_*PRE_TA*_ = 66.1 ± 5.9 r/min, PC_*POST_TA*_ = 67.1 ± 8.4 r/min, PC_*PRE_VL*_ = 68.7 ± 5.3 r/min, PC_*POST_VL*_ = 66.1 ± 8.6 r/min, PC_*PRE_ GM*_ = 65.9 ± 10.3 r/min, PC_*POST_GM*_ = 66.3 ± 10.7 r/min), so that any PRE–POST-1 difference in muscle activation and timing is not a cadence-driven effect.

### Effect of Biofeedback on Target Muscles

The delay between the POST-1 and PRE trial for TA, VL, and GM muscles across all subjects were 56.3 ± 27.0, 48.6 ± 27.2, and 42.2 ± 30.4 (mean ± SD) respectively, as shown in Table 1. Eight out of 10 subjects showed statistically significant changes in all target muscles. Two subjects (highlighted in light gray in Table 1) failed to significantly change the timing on at least one target muscle.

**TABLE 1:** Effectiveness of the biofeedback on the target muscles, expressed as the delay of the EMG envelopes between the POST-1 trial and PRE trial.

Subject #	TA (mean (SD)	VL (mean (SD)	GM (mean (SD)
1	12.09 ± 28.6	12.6 ± 19.0*	−2.2 ± 39.0
2	78.1 ± 22.8*	65.3 ± 32.5*	88.8* ± 41.3*
3	43.1 ± 21.4*	40.2 ± 26.1*	64.3* ± 17.2*
4	146.5 ± 44.5*	43.9 ± 33.6*	−158.0 ± 34.5*
5	48.1 ± 23.4*	68.8 ± 13.5*	61.4 ± 10.6*
6	82.3 ± 17.8*	71.4 ± 18.4*	66.6 ± 30.1*
7	−1.6 ± 46.0	−22.5 ± 36.7	80.5 ± 66.5*
8	14.4 ± 25.7*	43.3 ± 33.0*	47.8 ± 15.4*
9	29.6 ± 18.7*	36.0 ± 20.8*	77.5 ± 24.1*
10	110.2 ± 21.3*	127.1 ± 38.7*	94.9 ± 25.0*
Mean ± SD across all subjects	56.3 ± 27.0	48.6 ± 27.2	42.2 ± 30.4

*Values are expressed in degrees (°) and report circular mean ± circular standard deviation. Statistically significant values are indicated with an asterisk (p-value < 0.05 in the Watson–William parametric test). Only values from target muscles are reported.*

**FIGURE 3:**
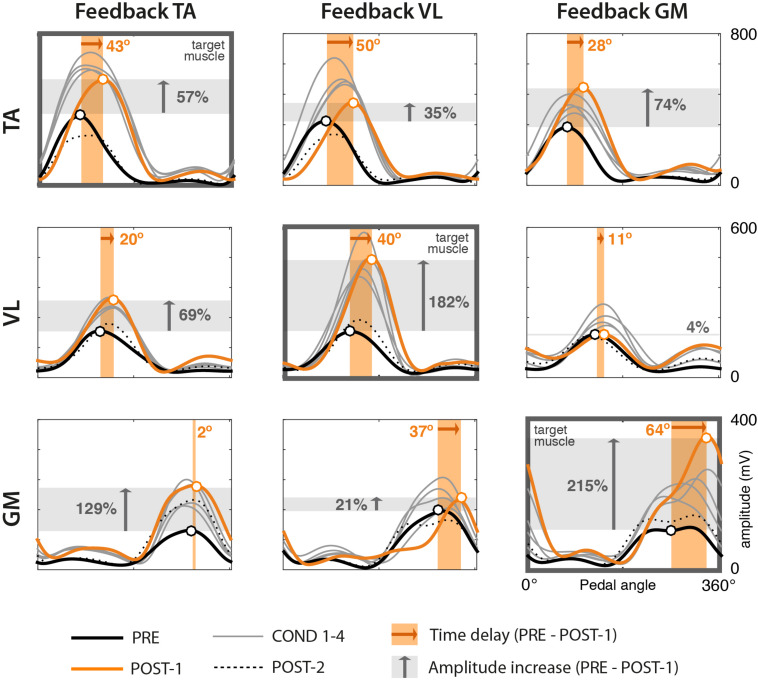
EMG envelopes from one representative subject (Subject 3) across trials. Rows represent the three muscles TA, VL, and GM. Columns represent the three different experimental sessions, each considering a different muscle for visual biofeedback (target muscle). Each curve represents the mean profile of the 60 normalized EMG envelopes of one trial. Trials correspond to the following color code. Bold black: PRE trial. Thin gray: COND 1–4 trials. Orange: POST-1. Dotted black: POST-2. Black and orange circles represent the mean peaks of the envelopes of the PRE and POST-1 trials, respectively. The width of the shaded orange area represents the mean delay between PRE and POST-1 trials (whose values are presented in Table 1). The height of the gray area represents the mean value of the amplitude difference (whose values are presented in Table 2).

Most of the subjects preserved the shape of EMG envelopes across the experiment. This is demonstrated by the high values of cross correlation between POST-1 and PRE trials, with mean values between 0.89 (TA) and 0.95 (VL) and standard deviation below 0.09. In some isolated cases, we observed lower similarities due to a change from a typical Gaussian-like shape to a double-peak waveform. This happened either in the PRE or POST-1 trial, but was never present on both trials. Figure 3 shows results on one representative subject (Subject 3) in terms of mean EMG envelopes, difference in delays (orange shaded area), and changes in amplitude (gray shaded area).

**FIGURE 4:**
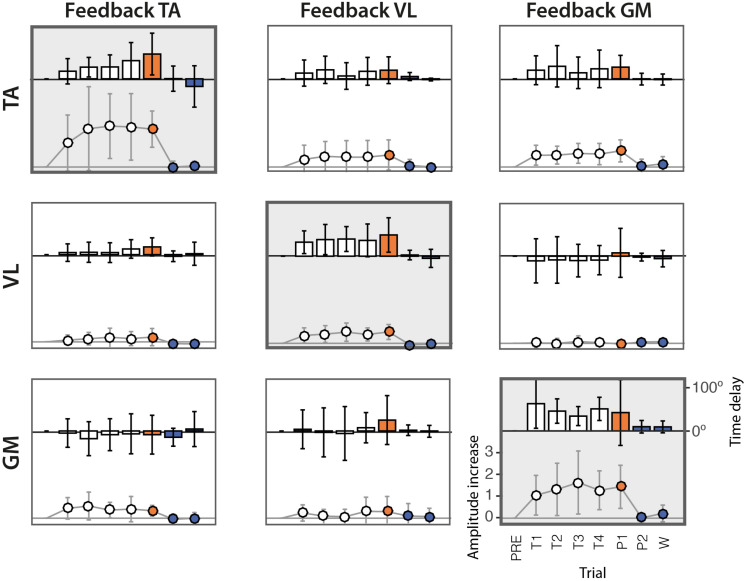
Delays and amplitude difference of the peak of EMG envelopes with respect to the PRE trial, across the 10 subjects. Rows represent the three muscles TA, VL, and GM. Columns represent the three different experimental sessions, each considering a different muscle for visual biofeedback. Bars represent the mean delays across subjects in degrees (scale shown on the right side). Solid lines represent the gain of the amplitude difference (scale shown on the left side). The vertical lines represent the standard deviation across subjects.

The analysis of the amplitudes (see Table 2) reveals that in the great majority of cases, the amplitude of EMG envelopes increased. The normalized amplitude difference for the target muscles TA, VL, and GM was 2.86 ± 1.15, 0.87 ± 0.47, and 2.40 ± 1.10, respectively. Figure 4 provides a compact representation of the mean delays and amplitude gains across all subjects. In this figure, it is also visible how the WASH trial (indicated as “W”) were in general effective to wash out the learning effects and make the EMG envelopes return to their initial conditions.

**TABLE 2:** Effect of the conditioning biofeedback on the amplitude of the EMG envelopes.

Subject #	TA (mean (SD)	VL (mean (SD)	GM (mean (SD)
1	1.74 ± 1.03*	0.10 ± 0.31	0.22 ± 0.31*
2	4.94 ± 3.06*	0.16 ± 0.34*	0.82 ± 0.61*
3	0.57 ± 0.29*	1.81 ± 0.66*	2.15 ± 0.83*
4	1.26 ± 0.76*	0.87 ± 0.33*	5.12 ± 1.51*
5	4.10 ± 1.04*	1.10 ± 0.50*	3.86 ± 0.95*
6	3.78 ± 1.18*	0.98 ± 0.47*	2.86 ± 1.21*
7	3.49 ± 1.40*	0.30 ± 0.25*	0.14 ± 0.80
8	2.29 ± 0.97*	1.37 ± 0.52*	2.10 ± 0.91*
9	3.48 ± 1.03*	1.38 ± 0.67*	3.81 ± 2.39*
10	2.95 ± 0.75*	0.64 ± 0.70*	2.89 ± 1.54*
Mean ± SD across all subjects	2.86 ± 1.15	0.87 ± 0.47	2.40 ± 1.10

*Values indicate the difference in peak amplitude between the POST and PRE trial, normalized with respect to the PRE trial mean amplitude (e.g., a value of 1 corresponds to a change in amplitude of 100%, meaning that the amplitude of POST peaks populations was two times the mean amplitude of the PRE trial). Statistically significant values are indicated with an asterisk (p-value < 0.05 in the t-test). Only values from target muscles are reported.*

**FIGURE 5:**
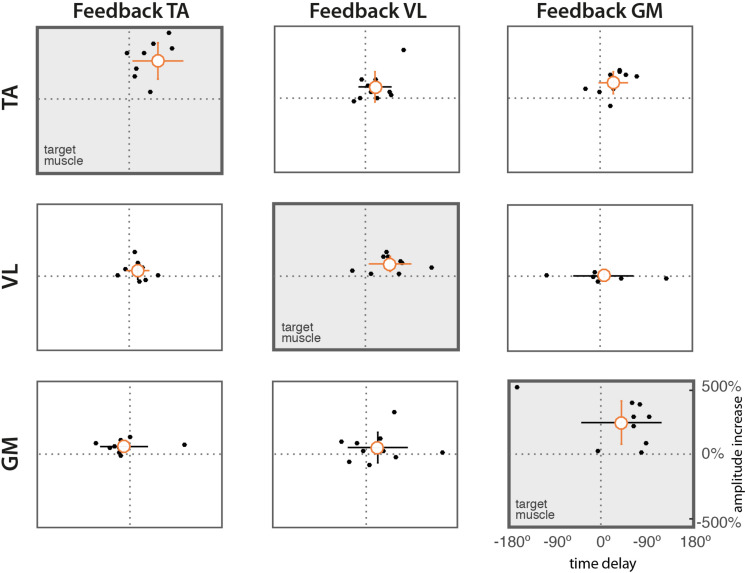
Scatter diagrams of time delays vs. difference in amplitude. Dots represent mean values from individual subjects. The orange circle indicates the mean value across subjects. Vertical and horizontal lines indicate the standard deviations of amplitude and delay, respectively. Lines in orange indicate means significantly different form zero (*t*-test, *p* = 0.05).

The scatter diagrams of Figure 5 show the relation between time delays and changes in amplitude for the 10 subjects. Results show a positive correlation between significant changes in amplitude and in time in the target muscles (diagonal subplots).

### Interaction Between Target and Non-target Muscles

Table 3 shows the comparison between the behavior of the target and the non-target muscles, for each subject and feedback session. We observed a general trend across subjects. When TA is the target muscle (session 1, first column for each subject), VL shows a similar positive delay with respect to the PRE trial, whereas GM shows an opposite, i.e., anticipated, activation. When VL is the target muscle (session 2, second column), TA shows a slight (not significant) positive relationship with VL. GM shows a more independent behavior, reporting both positive and negative delays across subjects. During the feedback of GM (session 3, third column), TA shows a positive correlation with GM, whereas VL appears to behave independently, showing very heterogeneous trends.

**TABLE 3:** Comparisons between target and non-target muscles of PRE-POST1 delays.

Subject		1			2			3			4			5			6			7			8			9			10			ALL		
Feedback session		TA	VL	GM	TA	VL	GM	TA	VL	GM	TA	VL	GM	TA	VL	GM	TA	VL	GM	TA	VL	GM	TA	VL	GM	TA	VL	GM	TA	VL	GM	TA	VL	GM
**Muscles**	**TA**	**+**	0	+	**+**	0	+	**+**	+	+	**+**	+	+	**+**	+	+	**+**	+	+	**0**	0	+	**+**	+	+	**+**	+	+	**+**	−	**+**	**+**	0	+
	**VL**	+	**+**	0	+	**+**	0	+	**+**	+	+	**+**	+	+	**+**	−	+	**+**	+	0	−	0	+	**+**	0	+	**+**	+	+	**+**	+	+	**+**	0
	**GM**	−	0	**0**	−	+	**+**	0	+	**+**	−	+	**+**	−	−	**+**	+	−	**+**	0	−	**0**	−	−	**+**	−	+	**+**	−	−	−	−	0	**+**

*For each subject, each column represents a specific feedback session, with the target muscles in the principal diagonal (in bold). The three symbols +, − or 0 indicate whether a specific muscle presents a positive, negative or non-significant delay between POST-1 and PRE trial. The last column represents the result across subjects, in which subjects not achieving significant values (“0” mark in the individual subject box) were excluded from the computation.*

### Synergy Analysis

**FIGURE 6:**
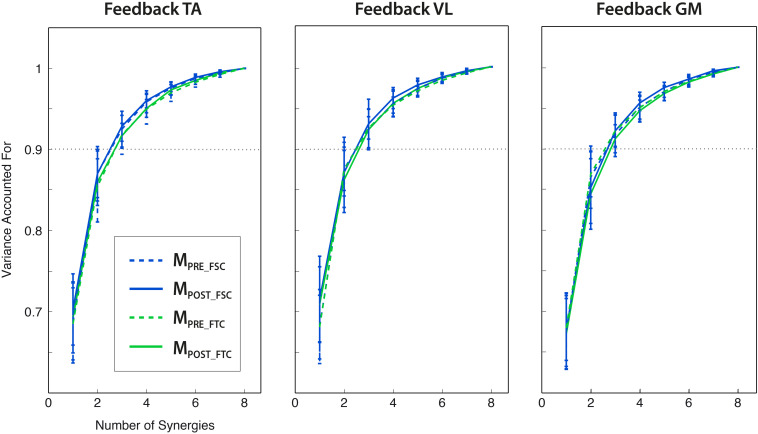
Variance explained by the extraction of a number of synergies from 1 to 8 (mean ± std across subjects), for different modular motor control models (FSC and FTC) and trials (PRE and POST). Three modules lead to the VAF > 0.9 criterion for all the analyzed conditions. All the reported VAF values have been calculated from synergies extracted from the training set.

**FIGURE 7:**
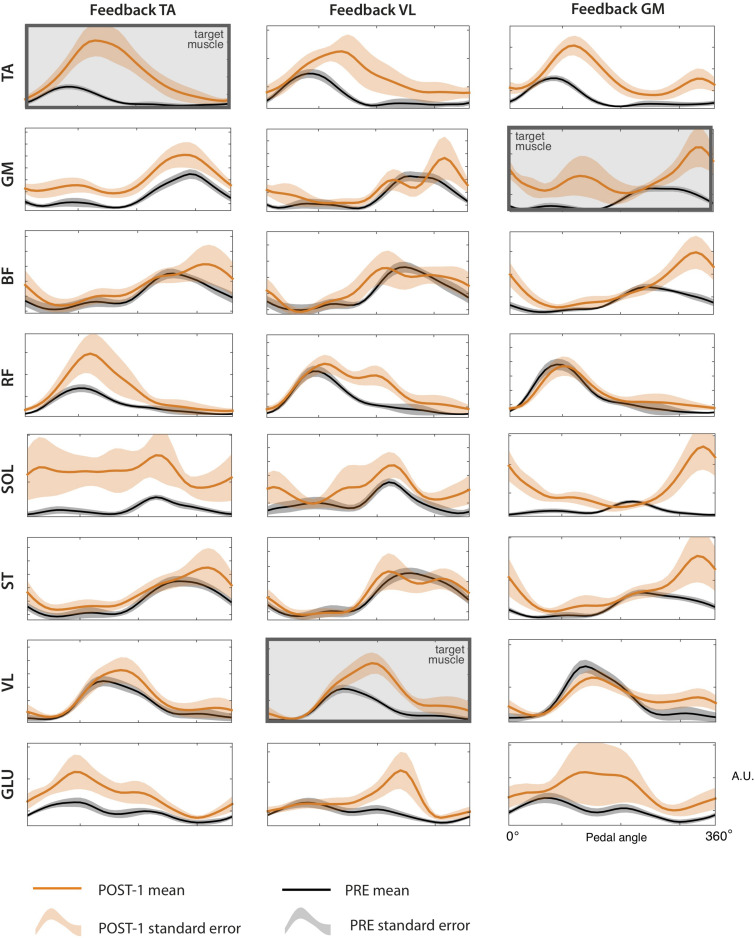
sEMG envelopes of all the recorded muscles for the each of the three biofeedback conditions, during the PRE and POST-1 trials. Data are represented as (mean ± standard error) across subjects.

By extracting muscle synergies from the training set for each subject, the dimensionality of the PRE and the POST-1 trials was three for all subjects, biofeedback conditions, and models (FSC and FTC), according to the VAF > 90% criterion, as shown in Figure 6. The grand average muscle activation across subjects for all the recorded muscles is shown in Figure 7, for each biofeedback condition.

#### Trial by Trial Extraction of Temporal and Spatial Synergies

**FIGURE 8:**
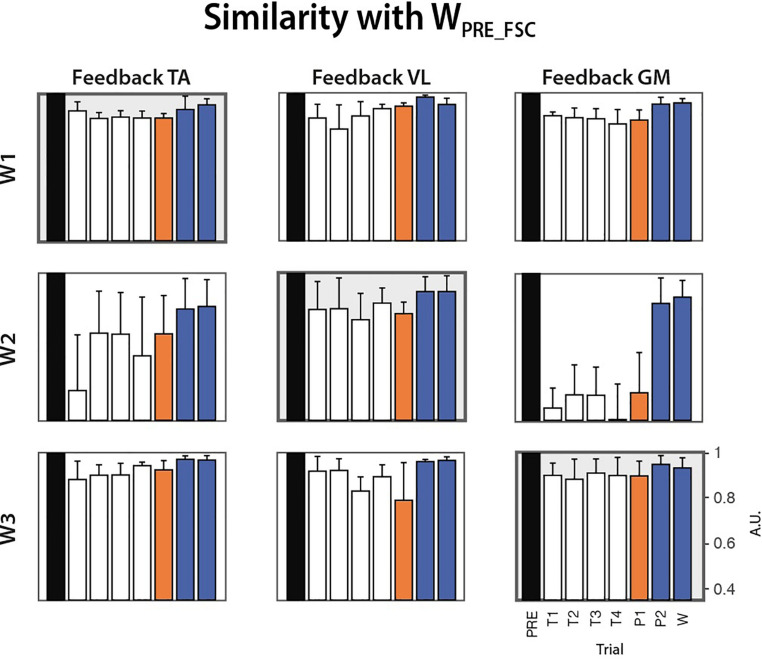
Cosine similarity between the synergies extracted from all the trials (COND1-4, POST1-2, WASH) and the homologous synergies extracted from the PRE trial for each biofeedback condition (median ± MAD across subjects).

Before testing the FSC and FTC models, we extracted synergies from all the trials (PRE, COND1-4, POST1-2, WASH) via NMF application, in order to characterize learning during the conditioning trials or the presence of any after-effects in the POST-2 and WASH trials. This was measured in terms of similarity between the W extracted at each trial and the corresponding one extracted at the PRE trial, for each subject and biofeedback condition. Figure 8 reports the evolution of this parameter along the trials. No clear monotonic learning curve is present. An abrupt change in the synergy structure is visible, especially W_2_ in the TA and GM feedback condition, starting from the first conditioning trial. No clear after-effect is present, as in POST-2 and WASH trials the similarity with the synergies extracted at the PRE trials is consistently higher than 0.8.

#### Comparison Among Modular Control Models

**FIGURE 9:**
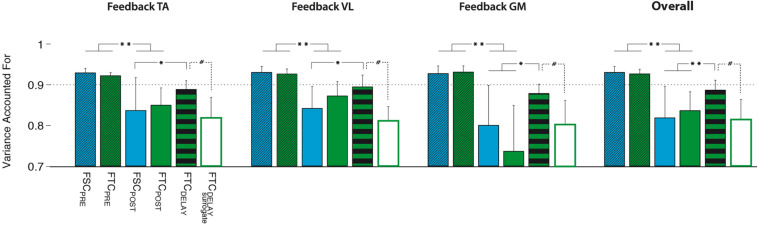
Performance of the different models, i.e., FSC (fixed W), FTC (fixed H), and FTC_*DELAY*_ (with the best performing surrogate), quantified in terms of variance accounted for (VAF) on the PRE and POST-1 testing sets. All models have been created using the 30 cycles of the M_*PRE*_ training set. Significance level was set according to *post hoc* analysis (**p < 0.002, *p < 0.02) and Wilcoxon signed rank test (#*p* < 0.05).

When testing the FSC model, we found that all the PRE trials were successfully reconstructed using W_*FSC_PRE*_ obtained from the training set (Figure 9, FSC_*PRE*_), with a VAF value higher than 90%. However, POST-1 trials reconstructed with W_*FSC_PRE*_ lead to VAF values lower than those obtained with FSC_*PRE*_ (*p* > 0.05) and even not different from the value expected from unstructured spatial components W_*FIX_SURR*_ (*p* > 0.05), meaning that the FSC model is not valid to describe this learning task.

When testing the FTC model (see Figure 9), the reconstruction of the M testing set of the PRE trial by using H_*FTC_PRE*_ led to VAF values typically higher than 90%, and with a value significantly higher than that expected from unstructured temporal commands H_*FIX_SURR*_ (*p* < 0.05). However, for the reconstruction of the POST-1 trial, H_*FTC_PRE*_ led to a significantly lower VAF as compared to the PRE trial, indicating that an unchanged temporal structure was not able to represent the observed changes in muscle coordination between PRE and POST-1. However, a significantly higher reconstruction VAF, comparable with that of the PRE trials of both FSC and FTC models, was obtained when using the FTC_*DELAY*_ model (see Figure 9, FTC_*DELAY*_).

For each biofeedback condition, the ANOVA test highlighted a significant effect of the model used to reconstruct the testing dataset. *Post hoc* analysis revealed that the VAF reconstruction value obtained via the FTC_*DELAY*_ model in the POST-1 trial is significantly higher than the one obtained with the FCT_*POST*_ and FSC_*POST*_, and it is not different from the VAF reconstruction values obtained on the PRE testing set via the FTC_*PRE*_ and FSC_*PRE*_ models for all the biofeedback conditions. Overall, the FTC model with optimal delay reached significantly higher reconstruction quality values when compared to the FSC and FTC models (see Figure 9, right panel).

#### Validation of the FTC_*DELAY*_ Model

The FTC_*DELAY*_ model properly captured the changes in muscle coordination observed in the POST-1 trial. This reconstruction was higher than that obtained by the surrogate data for the FTC_*DELAY*_ model, obtained by applying all the possible time shifts to the surrogate version of the temporal commands H_*FTC_PRE*_ (Figure 9). This makes the FTC_*DELAY*_ model the only one able to explain the changes in muscle coordination for all the biofeedback conditions.

**FIGURE 10:**
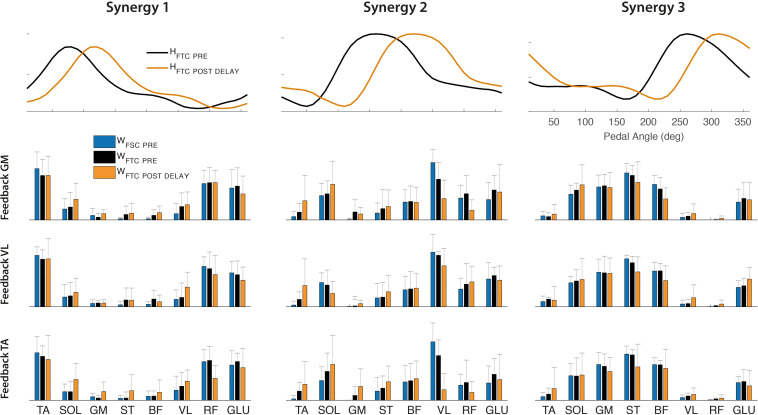
Upper panel: Optimal shift of the activation coefficients obtained by FTC_*DELAY*_ model (orange line) to reconstruct the POST-1 trial, compared to the original set of activation coefficients extracted by the PRE trial (black line). Lower panel: Synergy vectors obtained from the FTC_*DELAY*_ model on the POST-1 training set (orange bars), compared to those extracted on the PRE trial from the FTC model (black bars) and FSC model (blue bars).

The synergy vectors shown in Figure 10 (lower panel) obtained from H_*FTC_PRE*_ show substantial adjustments in the POST-1 trial (FTC_*DELAY*_ model), with cosine values among homologous pairs of synergy vectors reported in Table 4. In particular, the GM and TA biofeedback with the FTC_*DELAY*_ model determine a significant change in the structure of one synergy vector (W_2_), while few differences are present under VL feedback, indicating a tendency toward the preservation of the spatial components for this specific biofeedback condition.

**TABLE 4:** Average cosine of the angle between W related to the FTC **(A)** and FSC **(B)** model in the PRE trial and the corresponding homologous W emerging from the FTC_*DELAY*_ model in the POST-1 trial.

(A)	TA	VL	GM
W1_*FTC/*_W1_*FTC_DELAY*_	0.86 ± 0.09	0.82 ± 0.13	0.77 ± 0.17
W2_*FTC/*_W2_*FTC_DELAY*_	0.61 ± 0.24	0.77 ± 0.23	0.54 ± 0.22
W3_*FTC/*_W3_*FTC_DELAY*_	0.85 ± 0.15	0.85 ± 0.13	0.78 ± 0.19

**(B)**	**TA**	**VL**	**GM**

W1_*FSC/*_W1_*FTC_DELAY*_	0.88 ± 0.08	0.86 ± 0.14	0.79 ± 0.17
W2_*FSC/*_W2_*FTC_DELAY*_	0.61 ± 0.26	0.73 ± 0.26	0.38 ± 0.18
W3_*FSC/*_W3_*FTC_DELAY*_	0.85 ± 0.16	0.84 ± 0.12	0.78 ± 0.19

## Discussion

### Effectiveness of EMG Biofeedback

Results confirm our first hypothesis (*task feasibility*), demonstrating that conditioning exercises based on EMG biofeedback can promote changes in muscle timing during cycling. Most of the subjects (eight out of 10) were able to adjust the timing of muscle activation of all target muscles by means of a simple visual representation of the EMG envelope presented at the end of each pedaling cycle (Table 1 and Figure 3). In contrast, subjects showed a very heterogeneous behavior in terms of magnitude of the delays, suggesting that the adaptation strategy is strongly subject-specific (Figures 4, 5). However, as shown in Figure 5, we found that a significant change in delays is, in most subjects, accompanied by a significant increase in the amplitude of EMG envelope. It is unclear whether this is due to a physical (i.e., biomechanical) or a neural mechanism. Given the nature of the task, in which subjects were required to focus only on the timing while performing the cycling task, a change in the amplitude could be simply explained by the adoption of a different strategy. The shape of the EMG envelope in the target muscles did not change, with a Gaussian-like waveform in most cases, with some exceptions, in which the waveform presented double-peaks; this behavior could be due to compensation strategies, either related to the previously mentioned sub-optimal strategy or to a change in the biomechanical requirements during the pedaling cycle. When analyzing the behavior of non-target muscles, we observed significant time shifts in most cases. These results partly support our second hypothesis (*modular control*), showing that a change in one muscle activity is not limited to the target muscles, but involves the other muscles not used in the biofeedback loop. The direction of changes, either positive or negative, shows also some general trends (see Table 3). We observed a clear positive correlation between TA and VL, meaning that a positive delay in one of these two muscles is accompanied by a similar change in the other one. In contrast, GM seems to have a more independent behavior, in particular with respect to VL. Instead, VL and TA show a contradictory relationship depending on the muscle used for the biofeedback. These adjustments in timing in the non-target muscles are likely due to biomechanical constraints for the proper accomplishment of the pedaling task, in order to maintain an adequate stiffness at the lower limb joints in different parts of the pedaling cycle. However, this aspect needs to be further explored through the measurement of pedal forces and the calculation of the joint torque profiles with inverse dynamics.

### Spatial vs. Temporal Muscle Synergies

When testing the “modular control” hypothesis under the muscle synergy perspective, we observed that the modification in the individual muscle timing can be explained by some invariant modular control structures. Spatially fixed muscle synergies (FSC model) extracted during the PRE trial cannot account for the variability of the muscle activity from all the conditioning trials, indicating that learning a new strategy within the same task implies some reorganization of the spatial structure. In particular, changing the timing of a single muscle through biofeedback does not lead to coherent modification of the timing or amplitude of the original synergist muscles. Nevertheless, this learning task can be well explained by a modular control model with FTC shifted by an optimal delay (FTC_*DELAY*_ model). Under this hypothesis, a set of fixed temporal commands represents the variables that the CNS keeps fixed during a learning process, by adjusting only their timing and by differently weighting the contribution of each muscle to the overall coordination event-by-event. This latter model explains the learning task explored in this study, suggesting that an overall temporal invariance underlies a learning process. Preserving such a structure implies that the full-time course of the temporal recruitment throughout the cycle is maintained during the whole learning process. From a control point of view, this preservation of the temporal components is in line with existing theories of neural control of rhythmic tasks organized around a set of central pattern generators, shared across different tasks and adjusted in time to reflect different biomechanical requirements (Ivanenko et al., 2003, 2004; Cappellini et al., 2006). In general, the FTC models leads to a spatially variable structure, different from the one obtained through the FSC model. In particular, these adjustments are not general and appear to be dependent on the target muscle provided as biofeedback. However, this spatial alteration is strongly evident only for the synergy W_2_ under TA and GM feedback conditions, while W_1_ and W_3_ seem to preserve their original spatial composition under all biofeedback conditions. On the contrary, even though the FSC model does not account for the changes in muscle coordination under VL feedback, this condition has the tendency to preserve the spatial composition, as indicated by the higher similarity among the emerging spatial components.

When describing motor adaptation and motor learning within the muscle synergies framework, the modification in the module composition is in line with the hypothesis that the spatial (W) and temporal (H) parts of the modular organization sub-serve different neural mechanisms. Modifications to the temporal commands (H) can be used by the CNS for quick adjustments and corrections to already existing motor programs to adapt to external perturbation or to face different biomechanical demands. Instead, synergies (W) show a more slowly varying structure, whose change would imply a kind of permanent modification to the motor programs. This observation is in line with (Kargo and Nitz, 2003), showing that the tuning to already existing synergies allows for faster skill learning and with (Berger et al., 2013) showing that adaption to virtual surgeries is slower when new synergies are required. However, in our study, we found that after four conditioning trials, the subjects adjusted the timing of the target feedback muscle, but in order to do this they disrupted part of the spatial composition of muscle synergies, while keeping unchanged the shape of the temporal commands and adjusting their timing.

From the point of view of muscle coordination, the change in muscle activation timing might reflect both mechanical and neural constraints. Applying delayed muscle activation with respect to the pedaling cycle (phase shift) could be a pure biomechanical effect linked to changes in PC. This mechanism, known as activation dynamics, delays muscle activation when PC decreases in order to develop a constant force profile along the pedaling cycle, thus compensating for the fixed electromechanical delay of muscles (Neptune et al., 1997). In this study, we did not measure pedal force profiles, but the observed changes in cadence were not likely able to explain a pure effect of the activation dynamics mechanism. We thus assume that a neural component in the adjustment of timings was present during the learning process. From this point of view, the use of factorization algorithms for identifying adaptation strategies during this learning process could shed light on some basic neural mechanisms used by the CNS to face new biomechanical requirements; as a matter of fact, preserving a part of the original modular control scheme during a short-term learning process (either the spatial or the temporal part) could reflect the existence of habitual coordination patterns that leave aside any optimal control strategy (De Rugy et al., 2012). The adoption of such a habitual rather than optimal control scheme is further supported by the observed changes in amplitude during the POST-1 trials, indicating a tendency to find a solution which is good enough to face the current change in biomechanical requirements. Despite the habituality or optimality of the adopted motor control scheme, our task can be considered as quasi-constrained from a kinematic point of view, and the present experiment likely explores an extended (even though not complete) set of possible force outputs; in this scenario, the preserved modular control schemes are likely to be of neural origin (Kutch and Valero-Cuevas, 2012).

### Potential Applications in Neurorehabilitation and Limitations

One of the main functional consequences of a neurological injury is the reduced coordination complexity due to an incorrect timing of muscle activation. Cycling training is a technique used in neurorehabilitation to promote recovery of mobility-related functions, such as muscle strength, spasticity, cardiopulmonary function, and symmetry of movement (Katz-Leurer et al., 2006; Lee et al., 2008; Tang et al., 2009). Recent studies have shown that the combination of cycling exercises with visual and/or afferent stimulation improved walking and postural functions in neurological subjects (Ambrosini et al., 2011; Yang et al., 2014; Barbosa et al., 2015). Previous studies have also shown that a typical modular organization of cycling is present in healthy and neurological subjects (Raasch and Zajac, 1999; Hug et al., 2011; De Marchis et al., 2013a; Ambrosini et al., 2016; Barroso et al., 2016) with mechanisms similar to those underlying walking (Zehr et al., 2007; Barroso et al., 2014). These results provide preliminary evidence on the ability of cycling-based treatments to enhance the plasticity of the CNS, supporting its feasibility as a possible substitute of gait training after a neurological injury. Nevertheless, the effects of cycling-based training approaches on muscle coordination are still largely unexplored.

In the proposed experimentation, we limited our study to healthy people. Participants were asked to deviate from the normal pattern of muscle activation and execute movement in such a non-natural way, meaning that the resulting biomechanics might have not been functional. Whether a similar approach can be applied in a reverse fashion to restore coordination in people with neurological lesions should be investigated. The results obtained in this work let us hypothesize that, when disrupted muscle coordination is present, changing the timing of activation of a single muscle can have a functional result. This aspect has yet to be extensively explored, but it could lead to cycling-based rehabilitation programs based on feedback of small subset of internal variables.

A possible limitation of our study is the choice of visual biofeedback modality. There are multiple ways in which the feedback can be provided through visual indicators. These span from minimal visual cues based on binary states (Thompson and Wolpaw, 2015), to more complex feedback modalities based on multiple biological signals (Barbosa et al., 2015). We consider the EMG envelope a simple and sufficiently meaningful descriptor on the user’s physiological activity, which includes both time and shape information. To avoid the concomitant representation of the amplitude information, we decided to normalize the amplitude for each cycle, so that the user always saw a peak with unitary value. In pilot trials, this modality demonstrated to be more effective than including absolute amplitude information. We did not test whether the shape of the waveform was perceived as a distracting element. We nevertheless decided to leave such information, because it provided a qualitative indication of the correct execution of the task. In the future, we should investigate new forms of feedback such as those based on synergy analysis. For instance, substituting the EMG envelope with the activation coefficient from one synergy may be a feasible next step. One of the questions that we want to answer with this approach is whether “synergistic” feedback can produce better learning effects with respect to single muscle activity feedback. A negative response to this question will support the feasibility of this technique in clinical based context, where minimal experimental setups (one muscle instead of multiple muscle recording) can make the difference. Conversely, the eventual demonstration of a better effectiveness of the synergistic feedback will support the inclusion of muscle coordination as a valuable biomarker during re-learning approaches. These aspects are in our opinion relevant and worth being investigated.

In our experiment, the task goal was to postpone the peak of activation of the target muscle. Other task goals such as anticipating the peak timing, the amplitude, or accelerating/decelerating the movement in specific sub-phases of cycling are other options that can be considered in future studies. A main objective in this respect would be to develop look-up tables able to match each learning strategy with the resulting functional effects. The successful definition of these look-up tables will enable the definition of subject-specific rehabilitation programs. In this respect, it may be also interesting to establish which are the functional boundaries of each task goal, i.e., establishing to what extent can a subject change the target variable according to the musculoskeletal (e.g., leg-pedal kinematic chain, muscle dynamics) or neural (e.g., reaction time) constraints.

Even though different models of muscle coordination have been tested, additional insights onto this pedaling learning task could be provided by recording muscle activity from the non-dominant leg, in order to highlight potential compensation mechanisms adopted by the subjects. This can also be done by recording pedal forces, in order to gather information on the symmetry in task execution.

Future studies should possibly include the measurement of the most relevant joint kinematics of the subjects, e.g., ankle, knee and hip, together with the crank and pedal angle to have complete information on the biomechanical effects induced by this task. This may shed some light on the biomechanically vs. neural implications of muscle synergy adjustments during learning, which have not been specifically addressed by our work.

## Conclusion

In this study, we showed that muscle coordination during pedaling can be voluntarily changed through a conditioning procedure based on EMG visual feedback on one single muscle. We observed that changes in the target muscle timing are consistently accompanied by changes in other muscles not involved in the biofeedback loop. While changes in time and amplitude are in general subject-specific, they appeared to be correlated to each other, meaning that a shift in time is in general associated with a change in amplitude. Under the muscle synergy perspective, we showed that among the tested models, i.e., spatially and temporally invariant components, the one based on FTC (shifted in time) can better explain changes in muscle coordination. These results demonstrate that some underlying modular structures may be preserved even in the presence of significant changes in individual muscles. Our results also suggest that testing the effectiveness of only one of model (e.g., spatially fixed as typically done in literature) over surrogate data is not sufficient. Testing and compare alternative models may be key to identify the biomechanical or neural implications of the obtained results, especially for the applicability of synergy-based strategies in neurorehabilitation.

## Data Availability Statement

The datasets generated for this study are available on request to the corresponding author.

## Ethics Statement

The studies involving human participants were reviewed and approved by Departamento de Ética en la Investigación, Comité de Ética Consejo Superior di Investigaciones Científicas (CSIC). The patients/participants provided their written informed consent to participate in this study.

## Author Contributions

DT designed and executed the experiment, processed the data, discussed the results, and wrote the manuscript. CD processed the data, discussed the results, and wrote the manuscript. Ad’A processed the data, discussed the results, and made substantial intellectual comments during the revision of the manuscript. DNT executed the experiment and collected the data. FB and JP made substantial intellectual comments during the revision of the manuscript. All authors contributed to the article and approved the submitted version.

## Conflict of Interest

The authors declare that the research was conducted in the absence of any commercial or financial relationships that could be construed as a potential conflict of interest.
